# Pediatric In-Hospital Cardiac Arrest International Registry (PACHIN): protocol for a prospective international multicenter register of cardiac arrest in children

**DOI:** 10.1186/s12872-021-02173-5

**Published:** 2021-07-31

**Authors:** Jimena del Castillo, Débora Sanz, Laura Herrera, Jesús López-Herce, Grupo de Estudio de Parada Cardiaca Intrahospitalaria en la Infancia, Cristina Calvo, Carmen Campos, Sonia Cañadas, Juan Carlos de Carlos, Maria Concepción Goñi, Ramón Hernández, Ana Jordá, Juan Mayordomo, Abián Montesdeoca, Antonio Morales, Sara Moralo, Ana Muñoz, Aránzazu Olloqui, Antonio Rodriguez, María Luisa Serrano

**Affiliations:** 1 Pediatric Intensive Care Unit, Gregorio Marañón General University Hospital, Condado de Treviño 9, 28033 Madrid, Spain; 2 Gregorio Marañón Health Research Institute (IISGM), Madrid, Spain; 3 Maternal and Child Health and Development Research Network (REDSAMID), Institute of Health Carlos III, Madrid, Spain

**Keywords:** Pediatric cardiac arrest, Cardiac arrest, Resuscitation, In-hospital, Clinical registry

## Abstract

**Background and aims:**

Cardiac arrest (CA) in children is a major public health problem. Thanks to advances in cardiopulmonary resuscitation (CPR) guidelines and teaching skills, results in children have improved. However, pediatric CA has a very high mortality. In the treatment of in-hospital CA there are still multiple controversies. The objective of this study is to develop a multicenter and international registry of in-hospital pediatric cardiac arrest including the diversity of management in different clinical and social contexts. Participation in this register will enable the evaluation of the diagnosis of CA, CPR and post-resuscitation care and its influence in survival and neurological prognosis.

**Methods:**

An intrahospital CA data recording protocol has been designed following the Utstein model. Database is hosted according to European legislation regarding patient data protection. It is drafted in English and Spanish. Invitation to participate has been sent to Spanish, European and Latinamerican hospitals. Variables included, asses hospital characteristics, the resuscitation team, patient’s demographics and background, CPR, post-resuscitation care, mortality, survival and long-term evolution. Survival at hospital discharge will be evaluated as a primary outcome and survival with good neurological status as a secondary outcome, analyzing the different factors involved in them. The study design is prospective, observational registry of a cohort of pediatric CA.

**Conclusions:**

This study represents the development of a registry of in-hospital CA in childhood. Its development will provide access to CPR data in different hospital settings and will allow the analysis of current controversies in the treatment of pediatric CA and post-resuscitation care. The results may contribute to the development of further international recommendations.

*Trial register*: ClinicalTrials.gov Identifier: NCT04675918. Registered 19 December 2020 – Retrospectively registered, https://clinicaltrials.gov/ct2/show/record/NCT04675918?cond=pediatric+cardiac+arrest&draw=2&rank=10

## Background

Pediatric in-hospital cardiac arrest (IHCA) is a rare event with possible devastating consequences. It is considered a major public healthcare problem. Thanks to advances in cardiopulmonary resuscitation (CPR) guidelines and teaching skills, results in children have improved [1, 2]. However, pediatric CA still has a very high mortality [3–5].

Survival to pediatric IHCA has been related to multiple factors. Previous studies have described the influence of patient characteristics, quality of CPR, post-resuscitation factors and team interventions in mortality [3, 4, 6–8]. Current recommendations on CPR have insisted on the importance of quality and post-resuscitation care in an attempt to improve outcomes [9]. However, in the treatment of in-hospital CA there are still multiple controversies and gaps of knowledge.

### Quality CPR

Some clinical and experimental studies have suggested that quality of cardiopulmonary resuscitation (frequency and depth of chest compressions, compression-decompression ratio, coordination of chest compressions and ventilation with coordination of rescuers) is associated with better CPR results [10].

### Ventilation for pediatric CA

In the last decade, some authors have suggested that initial basic CPR with only chest compressions may be equal to or better than providing chest compressions plus ventilation. In contrast, clinical studies in children [11] and experimental studies in child animal models [12] have shown that early ventilation and oxygenation are essential in child CPR. Intubation during CPR has for many years been considered an essential maneuver during advanced CPR, as it achieves safe isolation of the airway and allows good ventilation. However, intubation is a technique that requires learning and training and can be difficult to perform in emergency situations such as a CA. In recent years, several studies in adults and children have suggested that intubation during CPR is associated with a worse survival and neurological prognosis [13, 14], but a recent large study in adults does not confirm these results [15]. There are no controlled, experimental, descriptive or clinical studies that have looked at this problem.

### Post-resuscitation factors

Once ROSC is achieved, efforts towards survival have to assess and treat post-cardiac arrest syndrome. Outcomes in terms not only of survival, but of good neurological outcome, depend on various factors, including hemodynamics, ventilation and oxygenation, temperature control, sedation and analgesia.

Hypotension after ROSC is associated with a worse prognosis. The ILCOR group recommends keeping systolic blood pressure (BP) above the 5th percentile, but there are no studies that assess which is the best BP in the post-resuscitation period or whether treating low blood pressure influences outcomes [16].

Several studies in adults and children have analyzed the influence of ventilation and oxygenation after ROSC on prognosis. Although the results between all the studies are not in agreement, most suggest that both hypoventilation and hyperventilation are associated with a worse prognosis [17]. Hypoxia can also be a poor prognostic indicator, while the results with hyperoxia are contradictory [16].

Hyperthermia after ROSC is common and is associated with a worse neurological prognosis, which increases with each degree of body temperature above 37 °C. Experimental studies in animals have demonstrated the neuroprotective effect of hypothermia. Initial studies in adults found that hypothermia improved the neurological prognosis in patients recovered from CA. However, the most recent studies in adults and children have found that therapeutic hypothermia is not associated with a better neurological prognosis or longer survival [18]. Therefore, at the present time, strict temperature control and avoiding hyperthermia are recommended.

Sedation and analgesia are fundamental in caring for the critical care child. They enable anxiety and pain treatment as well as shivering control for temperature management. However, after CA its use can difficult neurological assessment, and to date, no study has been able to describe the influence of different analgosedation protocols in neurological outcomes.

### Rational for the study

For the past years, the International Liaison Committee on Resuscitation (ILCOR) has endeavored its efforts in the identification of these gaps of knowledge and their resolution. There are few clinical data to verify the efficacy of CPR interventions in the pediatric population. Thus, the development of collaborative multicenter studies is needed. The objective of this study is to develop a multicenter and international registry of in-hospital pediatric cardiac arrest including the diversity of management in different clinical and social contexts. Participation in this register will enable the evaluation of the diagnosis of CA, CPR and post-resuscitation care and its influence in survival and neurological prognosis.

## Study aims

The objective of PACHIN is (1) to study the relationship between the characteristics of cardiopulmonary resuscitation (CPR) and outcomes, focusing on the influence of ventilation parameters (intubation or not during resuscitation and controlled or uncontrolled ventilation) on the recovery of spontaneous circulation, survival, and short- and long-term neurological prognosis of children with in-hospital cardiac arrest. (2) To study the relationship between post-resuscitation measures (especially ventilation, oxygenation, blood pressure, analgosedation and temperature) with survival and short- and long-term neurological prognosis in children with IHCA. (3) To create a permanent register of pediatric in-hospital cardiac arrest for European and Latinamerican countries.

## Methods and analysis

### Study design

This study is a multicenter, international, prospective observational registry.

### Setting

Patients will be enrolled by participating investigators from European and Latinamerican countries. All sites are susceptible of treating pediatric cardiac arrest patients. Participating hospitals differ in levels of care but are all able to submit their data to the study’s database.

### Patient elegibility

Inclusion criteria: all children aged 1 month to 18 years who suffer a CA in hospital. For the study, CA is defined as the absence of vital signs requiring at least one minute of chest compressions. Subsequent episodes of CA may be included for the same individual.

Exclusion criteria: Patients being treated with extracorporeal circulatory support (ECMO or ventricular assistance) at the time of CA. Patients who suffer a cardiac arrest and require ECMO for ROSC, after performing conventional CPR, will not be excluded. Duration of the data collection period 24 months.

### Recruitment

**Fig. 1:**
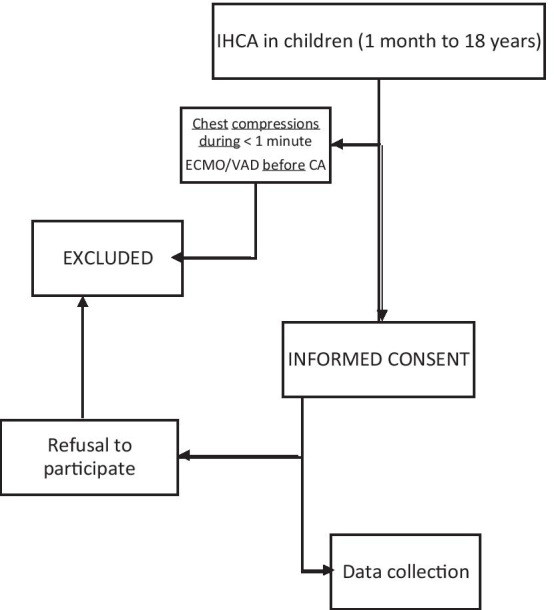
CONSORT (Consolidated Standards of Reporting Trials) flow diagram. IHCA: In-hospital Cardiac Arrest; ECMO: Extracorporeal Membrane Oxygenation; VAD: Ventricular Assistance Device

Study candidates will be identified by a study physician, who will explain the study to parents or guardians. Written informed consent will be obtained from parents or guardians prior to inclusion in the study. A CONSORT (Consolidated Standard of Reporting Trials) flow diagram is shown in Fig. 1.

### Data collection

Data will be collected, verified, and uploaded to a protected electronic web-based database (Xolomon) by their designated site coordinators/investigators under the oversight of their IRB. Database has been designed following the Utstein model [19]. Database is hosted according to European legislation regarding patient data protection. It is drafted in English and Spanish.

Variables recorded will include hospital characteristics, the resuscitation team, patient’s demographics and background, CPR, post-resuscitation care, mortality, survival and long-term evolution (see Table 1).

**Table 1:** Description of variables collected in database

Hospital characteristics: level of care; existence of cardiac arrest management plan; rapid response team: existence and composition; early warning scores
Patient demographics and characteristics: patient data before cardiac arrest (age, sex, weight, height, baseline neurological assessment, medical history, previous treatments)
Description of cardiac arrest: date and time; cause of arrest; place of arrest; cardiac rhythm; contributing factors
Description of cardiopulmonary resuscitation: resuscitation team (team members, number of providers, background); ventilation (airway management, ventilation objectives), chest compressions (rate, depth, synchrony, feedback device); defibrillation (shocks, energy); drugs (adrenaline, amiodarone, lidocaine, calcium, bicarbonate, fluids, others); quality control (invasive blood pressure, end tidal CO2); other treatments (ECMO, POCUS, drainage); time to ROSC; CPR ending; CPR complications; survival; cause of death
Post-resuscitation care: hemodynamic care (blood pressure, drug support, cardiac rhythm); ventilatory support (mechanical ventilation, ventilation parameters, oxygenation, blood gases results); neurological examination (BIS, pupils, Glasgow coma scale, POPC); renal support treatment; metabolic parameters; temperature control management
Outcomes: length of PICU and hospital stay; neurological outcome at discharge (POPC, Glasgow coma scale)
Long term follow-up: brain image (MRI, CT scan, EEG); neurological examination

### Outcomes assessment

The primary outcome is survival at hospital discharge. Secondary outcome being survival with favorable neurological status. Analysis of different factors influencing both outcomes will be performed.

### Statistical analysis

Data analysis plan: The data will be manually reviewed for errors, missing data and outliers before analysis. Extreme values will be set to missing if they are deemed unlikely, based on their validity range. Descriptive analysis of the data will be reported. Continuous variables will be reported as either median and interquartile range (IQR) or mean and standard deviation (SD) based on the distribution. Categorical var﻿iables will be described in numbers, percentages or both.

### Sample size

As this is an observational study with no intervention, we have not conducted a power calculation. Based on previous studies [3], we estimate that we will enroll at least 250 to 300 eligible cardiac arrest events. This would represent a large international dataset of pediatric cardiac arrest data with significant power to enable exploration of important correlations between performance and survival through multivariate analysis.

## Discussion

Multicenter collaborative studies are needed in order to assess the gaps in knowledge surrounding pediatric cardiac arrest. Our group as well as others, have developed collaborative platforms to this effect, demonstrating that this approach is feasible and useful [3, 20–22]. These experiences have identified those areas that need further assessment. This is the reason for the creation of a registry of in-hospital CA in childhood in European and Latinamerican countries. Its development will provide access to CPR data in different hospital settings. This data gathering from different countries and hospitals might enlighten the analysis of compliance with the actual guidelines in these countries and the identification of barriers in treatment of pediatric cardiac arrest. Multicenter international collaboration in Spanish and English will allow the investigators to recruit more patients and enable the analysis of current controversies in the treatment of pediatric CA and post-resuscitation care. We expect the results to contribute to the development of further international recommendations.

## Limitations

The main limitation of the study is that patient recruitment cannot be planned because CA is an unexpected sudden event. On the other hand, when CA happens, all attention is driven towards CPR, so it might be difficult to record all data related to the characteristics of CPR while performing it. Another limitation of the study is that the final results of CPR are influenced by many variables and it can be very difficult to differentiate the isolated effect of each factor. Furthermore, recording as many variables as aimed per CA requires a lot of dedication and careful review and supervision of each case by the study coordinator. Being CA a rare event in pediatric patients, it might be difficult to find statistically significant differences in some parameters such as mortality. Thus, the participation of a significant number of hospital centers and collaborating researchers is necessary. This is one of the fundamental reasons for a multicenter study. Finally, the neurological follow-up at one year of some patients will end after the end of the study period.

## Data Availability

Not applicable.

## Abbreviations

PACHIN: Pediatric In-Hospital Cardiac Arrest International Registry
CA: Cardiac arrest
CPR: Cardiopulmonary resuscitation
IHCA: In-hospital cardiac arrest
ROSC: Return of spontaneous circulation
ECMO: Extracorporeal circulatory support
ILCOR: International Liaison Committee on Resuscitation
IQR: Interquartile range
SD: Standard deviation
CONSORT: Consolidated Standard of Reporting Trials

## Declarations

### Trial status

Protocol version number and date: PACHIN 1, September 2020. Recruitment began he date recruitment began October 2020. Recruitment will be completed October 2025.
